# Impact of asymptomatic genital tract infections on *in vitro* Fertilization (IVF) outcome

**DOI:** 10.1371/journal.pone.0207684

**Published:** 2018-11-16

**Authors:** Susanna Ricci, Stefano De Giorgi, Elisa Lazzeri, Alice Luddi, Stefania Rossi, Paola Piomboni, Vincenzo De Leo, Gianni Pozzi

**Affiliations:** 1 Laboratory of Molecular Microbiology and Biotechnology (LA.M.M.B.), Department of Medical Biotechnologies, University of Siena, Siena, Italy; 2 Bacteriology Unit, Siena University Hospital, Siena, Italy; 3 Department of Molecular and Developmental Medicine, University of Siena, Siena, Italy; 4 Centre for Diagnosis and Treatment of Couple Sterility, Siena University Hospital, Siena, Italy; University of Padova, Medical School, ITALY

## Abstract

**Background:**

Infertility is estimated to affect approximately 9–30% of reproductive-aged couples. Several conditions involving one or both partners may contribute to infertility. The aim of this study is to evaluate the role of asymptomatic genital tract infections in the outcome of *In Vitro* Fertilization (IVF) in couples with infertility.

**Methods:**

A total of 285 infertile couples were enrolled in the study. Vaginal/endocervical swabs and semen samples were collected and subjected to microbiological analysis. Spermiograms were carried out on semen specimens, and lactobacilli were quantified in vaginal swabs. Data were associated with IVF results and analysed by using non parametric tests and multivariate analysis.

**Results:**

Microbiological analysis showed that 46.3% of couples presented with an asymptomatic genital tract infection. Spermiogram results showed a significantly diminished motility of sperm cells in samples positive to microbiological testing compared to negative specimens. *Enterococcus faecalis* was the most prevalent species (11.6%) in positive semen samples and was found to negatively affect both sperm morphology (*p* = 0.026) and motility (*p* = 0.003). Analysis of genital swabs from females showed that the presence of *E*. *faecalis* (*p*<0.0001), *Escherichia coli* (*p* = 0.0123), *Streptococcus agalactiae* (*p*<0.0001), and *Gardnerella vaginalis* (*p* = 0.0003) was significantly associated to reduced levels of vaginal lactobacilli. Association of microbiological data with IVF outcome showed that 85.7% of IVF+ couples was microbiologically negative, while IVF was successful in just 7.5% of couples infected with *E*. *faecalis* and/or *U*. *urealyticum* and/or *M*. *hominis* (*p* = 0.02).

**Conclusions:**

The results show the negative impact of *E*. *faecalis* on sperm quality and the association of definite bacterial pathogens with reduced levels of vaginal lactobacilli. The presence of *E*. *faecalis* and/or *U*. *urealyticum* and/or *M*. *hominis* in genital samples of infertile couples is predictive for a negative outcome of IVF.

## Introduction

Infertility is a medical condition that is appraised to affect between 9% to 30% of reproductive-aged couples worldwide [1]. In 2009, the International Committee for Monitoring Assisted Reproductive Technology (ICMART) and the World Health Organization (WHO) have defined ‘infertility’ as the failure to achieve a clinical pregnancy after 12 months of regular unprotected sexual intercourse [2]. Different pathological conditions affecting one or both partners may participate in infertility, including infections of the urogenital tract. Chronic or not appropriately treated infections are generally regarded as more critical for infertility compared to acute infections. Infections of the female genital tract may concern the vagina, the cervix, the uterus, or the tubal/pelvic area. Ascending infections are considered the most relevant for infertility as they can cause pelvic inflammatory disease and salpingitis which can eventually lead to tissue adhesions and tubal damage [3]. Male infections account for about 15% of total male infertility [3,4], leading to qualitative and quantitative sperm alterations [5]. Microbial pathogens present in semen can directly and indirectly impact on sperm quality and function [5]. Bacteriospermia may be accompanied by leukocytospermia, although its clinical relevance in male infertility is controversial [6–8].

Various genital pathogens have been implicated in infertility with different degrees of statistical significance. Infections caused by *Neisseria gonorrohoeae*, *Chlamydia trachomatis*, *Treponema pallidum*, human immunodeficiency virus (HIV), and mumps are relevant for infertility. In particular, *N*. *gonorrohoeae* can influence both male and female fertility, while *C*. *trachomatis* can affect sperm motility and viability, but it is particularly dangerous for the female where it can lead to tubal infertility [3,4,9]. Both Gram-positive and Gram-negative bacteria can alter sperm function. *Enterococcus faecalis* has been associated with oligozoospermia and teratozoospermia [5], whereas *Escherichia coli* has been shown to induce apoptosis in sperm cells and reduce their motility [5,10]. *Ureaplasma urealyticum* and *Mycoplasma hominis* are more commonly isolated from the genital tract of women and are considered as relevant for female infertility [3,11,12]. Nonetheless, both species were found to affect sperm motility and vitality [5,10,13]; additionally, *M*. *hominis* has been associated with abnormal sperm morphology [14] and *U*. *urealyticum* has been shown to damage nuclear chromatin with possible implications for embryo development [15]. On the contrary, neither *Mycoplasma genitalium* nor *Ureaplasma parvum* could be correlated to male infertility based on a recent meta-analysis [16], while *M*. *genitalium* has lately been appraised in relation to cervicitis and pelvic inflammatory disease [17].

The health status of the female genital tract is largely related to the presence of a normal vaginal microbiota [18]. The composition of the vaginal microbiota can profoundly influence all stages of female reproduction, starting from conception, throughout pregnancy until birth. Bacterial vaginosis (BV) is a vaginal microbiota disorder occurring when the normal flora, primarily composed of *Lactobacillus* spp., is reduced and replaced by mostly anaerobic microorganisms. BV is regarded as a dysbiosis of the vaginal microbiota and is associated to a heterogeneous cluster of pathogens rather than a single etiologic agent. The list of BV agents continues to enlarge and includes *Gardnerella vaginalis*, *Atobopium vaginae*, *M*. *hominis*, and different species of *Prevotella*, *Porphyromonas*, *Mobiluncus*, *Sneathia*, *Peptoniphilus*, *Anaerococcus* and *Clostridium* [19]. Recent epidemiological evidence indicate that BV may be sexually transmitted, suggesting that the male partner may serve as a reservoir for infection and re-infection [20]. Several studies have reported that BV is prevalent among infertile women, especially those with infertility due to tubal/pelvic factors [21]. In contrast, aerobic vaginitis (AV) has been described as an inflammatory condition in which a *Lactobacillus*-based microbiota shifts to a microbiota dominated by enterobacteria, staphylococci, streptococci, and enterococci [22]. These disorders of the normal vaginal microbiota have been associated to increased risk of miscarriage and preterm birth [21,23–25].

Assisted Reproductive Technology (ART) consists of all procedures that include *in vitro* handling of human oocytes and sperm cells or embryos with the purpose of establishing a pregnancy [2]. Among the *in vitro* fertilization (IVF) approaches, introduction of the intracytoplasmic sperm injection (ICSI) procedure in the early 1990s represented a major breakthrough in reproductive medicine. ICSI, initially preferred to other techniques to overcome male factor infertility, is now also employed for advanced maternal age and idiopathic infertility. Currently, ICSI reckons for approximately 70–80% of total ART cycles, thus representing the most commonly used ART treatment [26].

Despite the fact that genital tract infections are recognized to affect human fertility, there are still no consensus guidelines available on the microbiological management of infertile couples undergoing IVF treatment. In the present study, 285 infertile couples were tested for the presence of asymptomatic infections of the genital tract before being subjected to IVF, and results were associated with the outcome of IVF.

## Materials and methods

### Patients

A total of 285 couples, consecutively attending for 3 years the Centre for Diagnosis and Treatment of Couple Sterility at Siena University Hospital, were enrolled in the study. All couples presented with fertility disorders and were subjected to different medical examinations prior to undergoing IVF procedures. Among the medical tests, the presence of genital tract pathogens in semen and vaginal/endocervical swabs and the quality of semen were evaluated. None of the couples had signs or symptoms of genital infection. Written informed consent was obtained from each patient. The local ethical committee CEAVSE (Comitato Etico Area Vasta Sud Est) approved conduction of the study.

### Samples

Semen and vaginal/endocervical specimens were obtained about two months before IVF procedure. Semen was collected by masturbation after 3–5 days of sexual abstinence and subjected to spermiogram according to WHO guidelines [27]. Male patients were given instructions to perform semen collection after accurate genital hygiene and discard of urine first void. For each female patient, both a vaginal and an endocervical swab were collected using sterile cotton swabs (FL Medical, Padova, Italy). All samples were sent to the laboratory of clinical microbiology for analysis.

### Detection and identification of cultivable pathogens

Standard bacteriological culture methods were used to detect genital pathogens from semen specimens and vaginal swabs. Semen was used directly upon arrival at the laboratory, while vaginal swabs were immersed in 1 ml of saline solution (0.9% NaCl) for 15 min prior to testing. Vaginal swabs were employed to assess for the presence of vaginal pathogens along with lactobacilli (see below). Selective and differential solid media (Oxoid, Milan, Italy) were used for cultivable microorganisms, including gram-positive cocci, gram-negative bacteria, lactobacilli, anaerobes, and fungi. Species identification was carried out by using Matrix Assisted Laser Desorption Ionisation-Time Of Flight (MALDI-TOF) VITEK MS (Biomérieux Italia S.p.A., Florence, Italy) coupled with the Myla software v2.0 with a cut-off identification ≥ 99%. Semen samples were considered positive if bacterial concentrations were ≥ 5x10^3^ cfu/ml according to WHO guidelines recommending 10^3^ cfu/ml as the cut-off value for ‘significant bacteriospermia’ [27,28]. Vaginal swabs were regarded as positive at viable counts ≥ 10^5^ cfu/swabs.

### Vaginal lactobacilli

Quantitative analysis of lactobacilli in vaginal swabs was carried out on selective media for lactobacilli (Rogosa agar, Oxoid) and anaerobes (Schaedler agar, Oxoid). Presence of lactobacilli was regarded as ‘normal’ when cfu counts were ≥ 10^4^ cfu/swab, and ‘low’ at values < 10^4^ cfu/swab. Assay detection limit was 10^2^ cfu/swab.

### Detection and identification of non-cultivable pathogens

Genital tract pathogens with fastidious growth requirements or non-cultivable, including *C*. *trachomatis*, *U*. *urealyticum*, *M*. *hominis*, *N*. *gonorrhoeae*, *Trichomonas vaginalis* and HSV, were searched out both in semen and vaginal/endocervical swabs by Real-Time PCR. Endocervical swabs were the specimen of choice for searching *U*. *urealyticum*, *C*. *trachomatis* and *N*. *gonorrhoeae*, whereas vaginal swabs were assessed for the presence of *M*. *hominis*, *T*. *vaginalis* and HSV. As abovementioned, swabs were immersed in 1 ml of saline solution (0.9% NaCl) for 15 min prior to analysis. All genital samples were heat-inactivated (85°C for 10 min) and subjected to automated DNA extraction using the MagNA Pure LC DNA Isolation Kit III (Roche Diagnostics GmbH, Mannheim, Germany) and the MagNA Pure LC machinery (Roche Diagnostics) according to the manufacturer’s instructions. For PCR reactions, 2 μl of DNA, 10 pmol of each primer (S1 Table) and 5 pmol of TaqMan probe (Roche Diagnostics) were used. Samples were transferred into a 96-multiwell plate (Roche Diagnostics), placed in the Light Cycler 480 (Roche Diagnostics) and programmed for 40 cycles of amplification (denaturation at 95°C for 15 sec, annealing and extension at 60°C for 1 min). Samples were considered positive with an average C_T_ value of 38. The initial target copy number in clinical samples was determined based on external standard curves specific for each microorganism. Standard curves generated by 10-fold dilutions of control DNA were linear over a range of 5 log units with an efficiency of 1.747 and a slope of -4.129. Detection limits were between 10 and 10^3^ target copies/μl of sample, depending on the efficiency of primer pairs and Taqman probe and the quality of biological samples.

### Semen analysis

Specimens were analysed according to WHO guidelines [27]. After liquefaction of the ejaculate, sperm concentration (number of sperm cells/ml), progressive and total motility were determined. Eosin Y test was used to detect necrotic sperm cells. Morphological examination of the specimens was carried out by counting 200 spermatozoa/sample and evaluating morphological abnormalities of sperm organelles (nucleus, acrosome, tail). Values of concentration, motility and morphology of sperm cells were considered altered if placed below the fifth percentile of the reference population [27]. Samples were evaluated as leukocytospermic from counts of 10^6^ leukocytes/ml of semen, according to WHO [28]. Leukocytes in semen specimens were counted by using the method of Politch [29]. Briefly, H_2_0_2_ (0.0375%) was added to 4 ml of benzidine stock solution (0.0125% in 50% ethanol, w/v; Sigma-Aldrich, Milano, Italy). Ten μl of seminal fluid were mixed with 20 μl of freshly prepared benzidine-H_2_O_2_ solution. After 5 min, 160 μl of phosphate buffered saline (PBS) were added, and peroxidase-positive (round brown cells) and peroxidase-negative (unstained) cells were counted using a Mackler chamber and a phase-contrast microscope.

### IVF procedure

Multiple follicle growth was obtained by using recombinant Follicle Stimulating Hormone (rFSH) at a dose of 150–300 IU based on the ovarian response as evaluated by hormone serum levels and ultrasound examination. As soon as the dominant follicle reached 14 mm in diameter, a Gonadotropin Releasing Hormone (GnRH) antagonist was administered daily. Ovulation was induced by injection of human chorionic gonadotropin (hCG) when at least three follicles of size > 16 mm were present in the ovaries. Oocyte pick-up was scheduled 34–36 h after hCG injection. Oocytes were fertilized by the ICSI procedure, and embryo transfer (ET) was performed 3–5 days after IVF. Serum levels of hCG were determined at day 14 post-ET, and the presence of a gestational sac was evaluated by transvaginal ultrasound examination at week 7 of gestation.

### Statistical analysis

Statistical analysis was performed using the software GraphPad Prism 5.0. Analysis of microbiological results was carried out by evaluating each infectious agent as a distinct unit, except for *Candida* species and *Enterobacteriales* other than *E*. *coli* which were analysed as two microbial groups. Results of spermiogram and microbiological analysis of semen samples were analysed by using the Mann-Whitney test. The Chi square (χ^2^) test (with Yates’ correction or Fisher exact test) was employed to assess the association between the presence of genital pathogens and vaginal lactobacilli in vaginal/endocervical swabs. The association of genital tract pathogens in infertile couples with the outcome of IVF was analysed by using the χ^2^ test (with Yates’ correction or Fisher test). Multivariate analysis was chosen to identify single infectious agents or groups associated as independent risk factors to IVF outcome. A *p* value lower than 0.05 was regarded as significant.

## Results

In this study, 285 infertile couples were tested for the presence of genital tract pathogens prior to undergoing IVF treatment. Microbiological analysis was conducted on a total of 855 genital samples, of which 285 semen specimens, 285 vaginal swabs and 285 endocervical swabs. A total of 195 clinical strains belonging to 25 different microbial species was detected in the samples tested (Table 1). *E*. *faecalis* represented the most common finding with a prevalence of 24.1% (47/195). Other frequently identified microbial species included *S*. *agalactiae* (15.9%), *E*. *coli* (15.4%), *M*. *hominis* (10.8%), *Candida* spp. (8.2%), and *U*. *urealyticum* (5.1%) (Table 1). Co-presence of two different pathogens was detected in 14 semen specimens and in 16 vaginal/endocervical swabs, while the simultaneous presence of 3 pathogens was observed in 3 samples. It should be noted that neither *C*. *trachomatis* nor *N*. *gonorrhoeae* were found in genital tract samples.

**Table 1:** Prevalence of microbial species in genital tract samples from infertile couples.

Microbial species ^a^	Genital tract samples ^b^		
	Semen	Vaginal/Endocervical swab	Total
*E*. *faecalis*	33	14	47
*S*. *agalactiae*	13	18	31
*E*. *coli*	19	11	30
*M*. *hominis*	3	18	21
*Candida* spp.	5	11	16
*U*. *urealyticum*	6	4	10
Other *Enterobacteriales*	5	4	9
*S*. *aureus*	2	4	6
*S*. *haemolyticus*	6	0	6
Group F *Streptococcus*	5	1	6
*G*. *vaginalis*	0	5	5
*T*. *vaginalis*	1	3	4
*P*. *asaccharolyticus*	2	0	2
*P*. *aeruginosa*	1	0	1
HSV	1	0	1
*Chlamydia trachomatis*	0	0	0
*Neisseria gonorrhoeae*	0	0	0
**Total**	102	93	195

^a^ *Candida* spp., *C*. *albicans*, *C*. *parapsilosis*, *C*. *glabrata* and *C*. *krusei*; other *Enterobacteriales*, *K*. *pneumoniae*, *K*. *oxytoca*, *P*. *mirabilis*, *M*. *morganii*, *E*. *aerogenes*, and *C*. *kroserii*.

^b^ A total of 855 genital samples (285 semen specimens, 285 vaginal and 285 endocervical swabs) from 285 males and 285 females were subjected to microbiological analysis. Both a vaginal and an endocervical swab were collected from each female patient. Endocervical swabs were used to search for *U*. *urealyticum*, *C*. *trachomatis* and *N*. *gonorrhoeae*, whereas vaginal swabs were employed to test for all other pathogens.

### Prevalence and aetiology of genital tract infections in infertile couples

**Fig 1:**
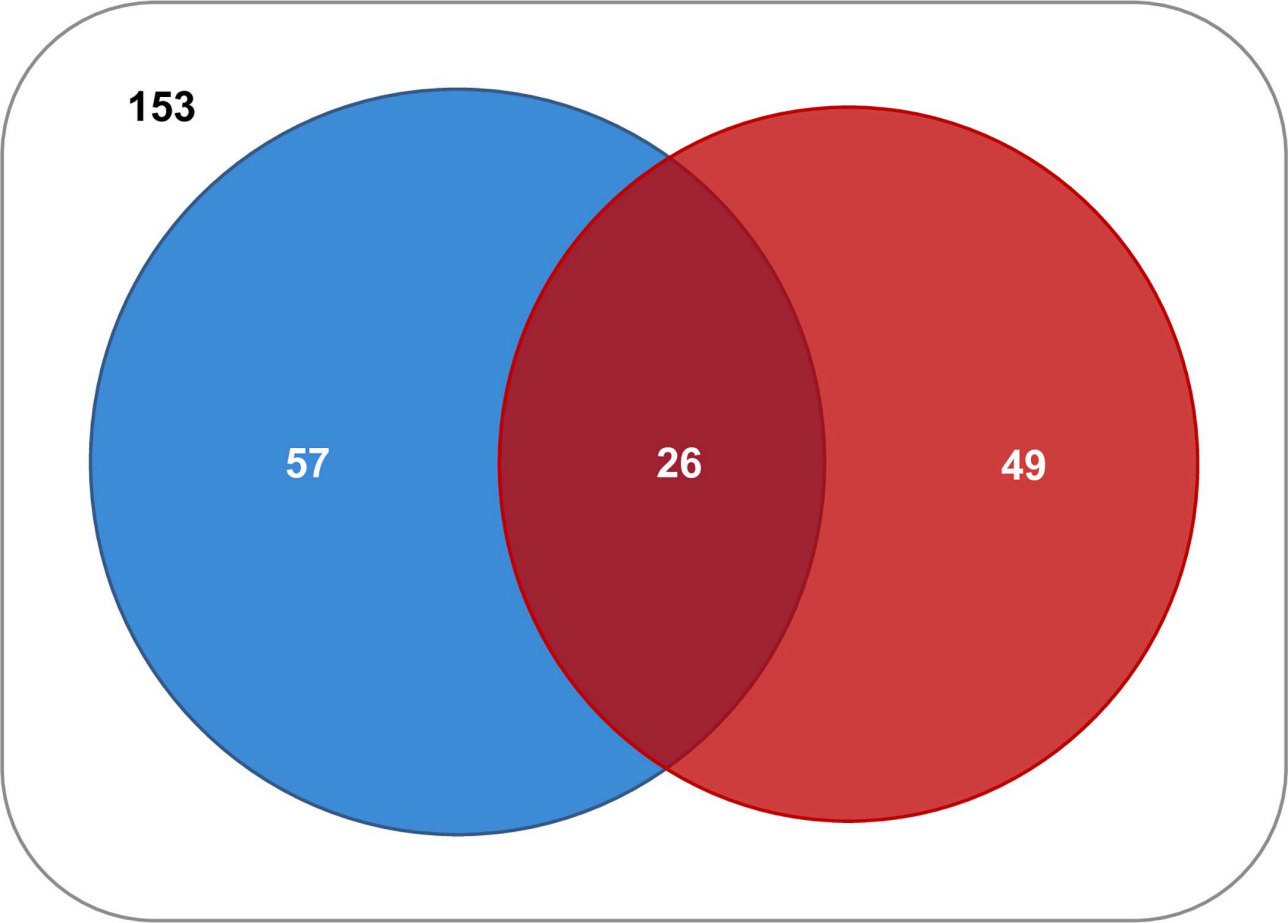
Venn diagram of genital tract infections in infertile couples. A total of 285 infertile couples were enrolled in the study and subjected to microbiological analysis to search for genital tract pathogens in semen specimens and vaginal/endocervical swabs prior to IVF. One hundred and thirty-two (46.3%) couples were positive for at least one pathogen in one or both partners. The male was positive in 57 cases (20%; blue) and the female in 49 cases (17.2%; red). The intersection of blue and red regions represents the couples (n = 26; 9.1%) where both partners were positive for at least one genital tract pathogen. The external set comprises all the couples included in the study.

**Fig 2:**
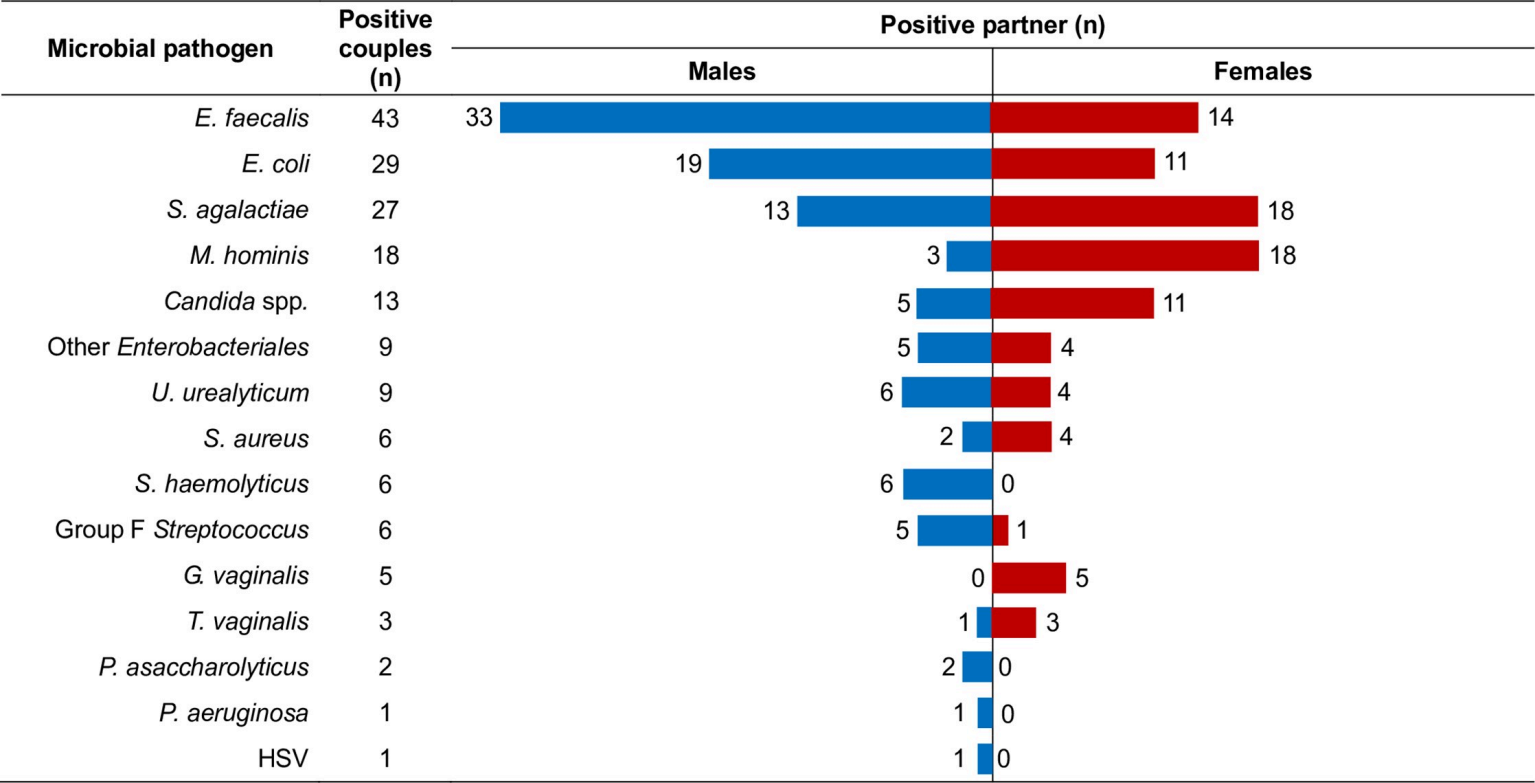
Prevalence of genital tract pathogens in infertile couples. Tornado graph showing the number of clinical strains of different microbial pathogens found in genital tract samples from male (blue bars) and female (red bars) patients. For each pathogen, the number of positive couples is also shown. Pathogens are listed according to their prevalence in infected couples. *Enterobacteriales* other than *E*. *coli* included *Klebsiella pneumoniae*, *Klebsiella oxytoca*, *Proteus mirabilis*, *Morganella morganii*, *Enterobacter aerogenes*, and *Citrobacter kroserii*. *Candida* spp. included *C*. *albicans*, *C*. *parapsilosis*, *C*. *glabrata*, and *C*. *krusei*.

Microbiological results showed that 29.1% (83/285) of males and 26.3% (75/285) of females were found positive to at least one genital pathogen. A total of 132 couples (46.3%) was positive for at least one pathogen in at least one of the two partners. Of the 132 positive couples, the male partner only was positive in 57 cases, the female only was positive in 49, and both partners were positive in 26 couples (Fig 1). Out of these 26 couples, 16 shared at least one genital pathogen between partners. The most prevalent microbial pathogens found in infected couples included *E*. *faecalis* (32.6%), *E*. *coli* (22%), *S*. *agalactiae* (20.5%), *M*. *hominis* (13.6%), *Candida* spp. (9.8%), *Enterobacteriales* other than *E*. *coli* (6.8%), and *U*. *urealyticum* (6.8%) (Fig 2). *M*. *hominis* and *T*. *vaginalis* were more prevalent in females, *G*. *vaginalis* was not found in males, and *S*. *haemolyticus* was not detected in females (Fig 2).

### Impact of genital tract pathogens on semen quality

A total of 285 semen specimens were analysed, of which 12 samples were azoospermic. Out of 273 samples, 72 (26.4%) had oligozoospermia (concentration < 15x10^6^/ml), 65 (23.8%) showed asthenozoospermia (total motility < 40%), 62 (22.7%) exhibited leukocytospermia (leukocyte counts ≥ 10^6^ cell/ml) and 28 (10.3%) presented with teratozoospermia (typical morphology < 4%). Percent sperm motility in samples with leukocytospermia (total motility = 46±17; progressive motility = 43±18) was significantly lower compared to that of specimens with normal counts of seminal leukocytes (total = 56±17; progressive = 53±18), (*p* = 0.0003).

Spermiogram results showed that both total (*p* = 0.012) and progressive (*p* = 0.0098) motility were significantly diminished in samples positive to microbiological testing compared to negative specimens (Table 2). Out of all pathogens identified, only *E*. *faecalis* was found to significantly alter semen parameters. Concentration, motility, and typical morphology of sperm cells were reduced in *E*. *faecalis*-positive compared to *E*. *faecalis*-negative samples (Table 2). Differences in total (*p* = 0.005) and progressive (*p* = 0.003) motility and in typical morphology (*p* = 0.026) of sperm cells between semen cultures positive and negative for *E*. *faecalis* were statistically significant (Table 2). Out of 62 samples with leukocytospermia, 49 (79%) were microbiologically negative (Table 3). Although leukocytospermia was higher in negative (25.8%) than in positive (15.7%) semen samples, differences were not significant (Table 3).

**Table 2:** Correlation of genital tract pathogens with semen parameters in infertile males ^a^.

Microbial pathogen (n) ^b^	Concentration (sperm cells/ml)	Motility (%) ^c^		Typical morphology (%)
		Total	Progressive	
*E*. *faecalis* (33)	4.03 x10^7^ ± 7.43 x10^6^	44.9 ± 3.1 **	40.3 ± 3.3 **	11.5 ± 1.4*
*E*. *coli* (19)	5.01 x10^7^ ± 1.32 x10^7^	49.5 ± 4.0	44.8 ± 4.5	16.2 ± 2.0
*S*. *agalactiae* (13)	5.19 x10^7^ ± 9.01 x10^6^	46.9 ± 6.1	44.4 ± 6.3	12.0 ± 2.1
*M*. *hominis* (3)	9.03 x10^7^ ± 1.05 x10^7^	54.3 ± 8.8	53 ± 8.6	15.9 ± 1.7
*Candida* spp. (5)	1.97 x10^7^ ± 1.31 x10^7^	45.4 ± 5.6	41.4 ± 8.2	11.8 ± 4.1
Other *Enterobacteriales* (5)	4.23 x10^7^ ± 1.53 x10^7^	41.0 ± 8.4	38.8 ± 8.2	12.1 ± 3.3
*U*. *urealyticum* (6)	9.13 x10^7^± 2.02 x10^7^	60.5 ± 6.9	59.8 ± 6.5	20.4 ± 5.1
*S*. *aureus* (2)	5.15 x10^7^± 9.50 x10^6^	55.5 ± 0.5	54.5 ± 0.5	14.0 ± 7.0
*S*. *haemolyticus* (6)	2.39 x10^7^± 1.39 x10^7^	39.5 ± 9.1	37.5 ± 7.8	11.3 ± 1.7
Group F *Streptococcus* (5)	2.28 x10^7^± 9.81 x10^6^	40.2 ± 9.1	35.6 ± 9.7	11.5 ± 3.4
*T*. *vaginalis* (1)	5.00 x10^7^	86.0	81.0	14.0
*P*. *asaccharolyticus* (2)	6.80 x10^7^ ± 3.80 x10^7^	66.5 ± 18.5	66.5 ± 18.5	22.5 ± 1.5
*P*. *aeruginosa* (1)	7.30 x10^7^	61.0	55.0	10.5
HSV (1)	3.00 x10^7^	54.0	50.0	12.6
**Total Positive** (83)	4.44 x10^7^ ± 4.84 x10^6^	**47.6 ± 1.3** *	44.2 ± 1.4 **	13.6 ± 0.8
**Total Negative** (190)	5.49x10^7^ ± 3.73x10^6^	53.4 ± 1.28	50.4 ± 1.4	14.9 ± 0.6

^a^ All data are represented as mean ± SEM.

^b^ Numbers (in brackets) of genital tract pathogens identified in 273 semen specimens. Twelve patients were excluded because azoospermic. Total positive (n = 83), number of males positive for at least one pathogen. Total negative (n = 190), number of males negative to microbiological testing.

^c^ Total motility, sperm cells moving in all directions; progressive motility, sperm cells moving along a straight line.

* Statistical analysis (Mann-Whitney test) was performed against the group of negative males. Significant differences are in bold: *, *p* < 0.05; **, *p* < 0.01.

**Table 3:** Association between the presence or absence of genital tract pathogens and leukocytospermia in semen samples.

Pathogens ^a^	Semen samples ^b^			*p* value ^c^
	Absence of leukocytospermia (%)	Presence of leukocytospermia (%)	Total	
Absence	141 (74.2)	49 (25.8)	190	0.09
Presence	70 (84.3)	13 (15.7)	83	
**Total**	211	62	273	

^a^ Microbial pathogens were searched in a total of 273 semen samples after exclusion of 12 specimens from azoospermic males.

^b^ Semen samples were considered as leukocytospermic when leukocyte counts were ≥ 10^6^/ml.

^c^ χ^2^ test with Yates’ correction.

### The presence of *E*. *faecalis*, *E*. *coli*, *S*. *agalactiae*, and *G*. *vaginalis* is associated to reduced levels of vaginal lactobacilli

**Fig 3:**
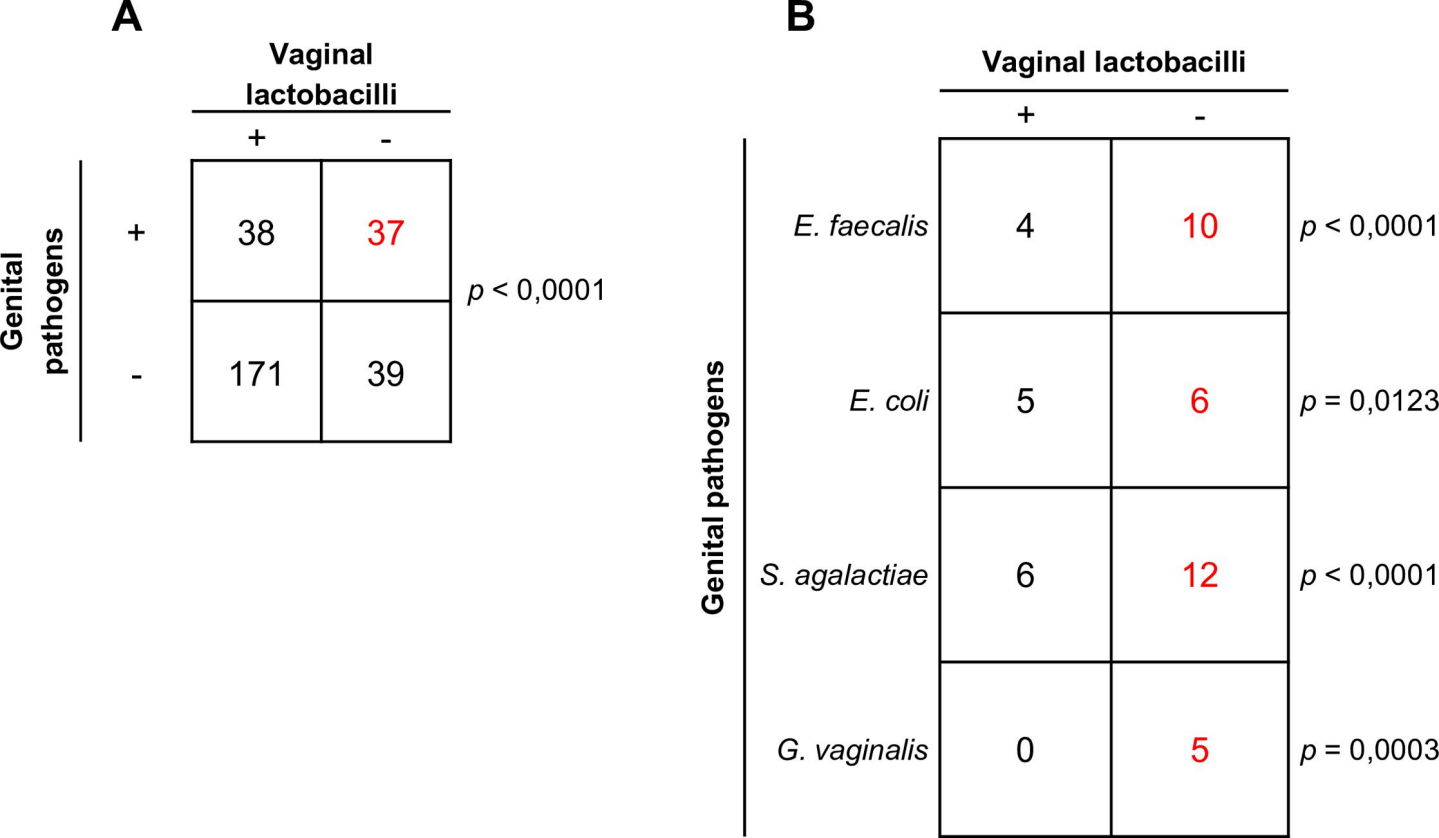
Association between genital tract pathogens and vaginal lactobacilli in infertile females. Both a vaginal and an endocervical swab were collected from each female patient enrolled in the study. Endocervical swabs (n = 285) were used to search for *U*. *urealyticum*, *C*. *trachomatis* and *N*. *gonorrhoeae*, while vaginal swabs (n = 285) were simultaneously tested for all other pathogens and vaginal lactobacilli. Lactobacilli were quantified on selective solid media, and counts < 10^4^ cfu/swab were regarded as reduced levels of lactobacilli. **A.** Contingency table reporting the number of swabs negative (-) or positive (+) for at least one genital tract pathogen in relation to the number of swabs with normal (+) or reduced (-) levels of lactobacilli. **B.** Contingency table showing the number of swabs positive for *E*. *faecalis*, *E*. *coli*, *S*. *agalactiae*, and *G*. *vaginalis* that presented with either normal (+) or low (-) levels of lactobacilli. The χ^2^ test with Yates’ correction was used for all cases except for *G*. *vaginalis* (Fisher exact test). For each of the above pathogen, statistical analysis was performed against the negative samples with normal (n = 171) or reduced (n = 39) levels of vaginal lactobacilli.

Genital swabs from 285 females were assessed for the presence of both genital tract pathogens and vaginal lactobacilli. A highly significant association (*p*<0.0001) between reduced amounts of vaginal lactobacilli (< 10^4^ cfu/swab) and the presence of genital tract pathogens was found in the female study population (Fig 3A). When analysis was applied to each individual pathogen, a statistically significant association was observed between reduced levels of lactobacilli and the presence of *E*. *faecalis* (*p*<0.0001), *E*. *coli* (*p* = 0.0123), *S*. *agalactiae* (*p*<0.0001), and *G*. *vaginalis* (*p* = 0.0003), (Fig 3B). It is worth noting that *G*. *vaginalis*, a key bacterial species of BV, was detected only in swabs with decreased load of vaginal lactobacilli.

### Infection with *E*. *faecalis* and/or *U*. *urealyticum* and/or and *M*. *hominis* is predictive of a negative IVF outcome in infertile couples

To investigate whether specific genital tract pathogens could be associated to IVF failure, microbiological results were correlated to IVF outcomes. IVF success was slightly higher in non-infected than in infected couples. Microbiological data indicated that specific pathogens (*E*. *faecalis*, *U*. *urealyticum*, *M*. *hominis*, *G*. *vaginalis*, *E*. *coli*) were more prevalent in unsuccessful (IVF-) than successful (IVF+) couples, however, no significant differences were calculated when each pathogen was tentatively associated with IVF outcome. Therefore, analysis was performed by examining couples positive for groups of genital tract pathogens after sequential exclusion of the pathogens that seemed not to affect IVF outcome. The microbial group constituted of *E*. *faecalis*, *U*. *urealyticum*, *M*. *hominis*, *G*. *vaginalis*, and *T*. *vaginalis* was more prevalent in IVF- than IVF+ couples, but differences were not significant (*p* > 0.05, χ^2^ test with Yates’ correction). Elimination of *T*. *vaginalis* showed that prevalence of the microbial group was significantly higher in IVF- (36.3%) compared to IVF+ (16.7%) couples (*p* = 0.03, χ^2^ test with Yates’ correction). Finally, by further excluding *G*. *vaginalis*, the smallest infectious group significantly associated with IVF failure included *E*. *faecalis* and/or *U*. *urealyticum* and/or *M*. *hominis* (Table 4). Analysis of the IVF+ couples showed that 30/35 (85.7%) were negative to microbiological testing, whereas out of the couples infected with *E*. *faecalis* and/or *U*. *urealyticum* and/or *M*. *hominis*, just 5/67 (7.5%) obtained a successful IVF (*p* = 0.02, χ^2^ test with Yates’ correction; Table 4). Interestingly, among the IVF- couples positive for this microbial group, *E*. *faecalis* and *U*. *urealyticum* were found in approximately 90% of cases, whereas *M*. *hominis* was detected in all the couples with a poor IVF outcome.

**Table 4:** Association between the presence of *E*. *faecalis* and/or *U*. *urealitycum* and/or *M*. *hominis* and IVF outcome in infertile couples.

Pathogens	IVF outcome			*p* value ^b^
	IVF- couples (%)	IVF+ couples (%)	Total	
Absence ^a^	111 (78.7)	30 (21.3)	141	0.02
*E*. *faecalis*, and/or *U*. *urealitycum*, and/or *M*. *hominis*	62 (92.5)	5 (7.5)	67	
**Total**	173	35	208	

^a^ Results refer to all the couples negative to microbial testing (n = 153) after exclusion of 12 couples where the male partner was azoospermic.

^b^ χ^2^ test with Yates’ correction.

## Discussion

Infertility is an ongoing challenge throughout the world and is increasingly being considered not only as a private matter but also as a public health burden. ART has allowed to overcome certain issues, however, rates of conception are still low [30]. Several factors can participate in infertility, including genital tract infections. However, no consensus guidelines are available yet on microbiological evaluation of infertile couples prior to undergoing IVF.

The present study focused on the impact of asymptomatic genital tract infections on couple fertility. Main findings are: (i) approximately half of the couples was diagnosed with a genital tract infection, (ii) *E*. *faecalis* had a significantly negative impact on sperm motility and morphology, (iii) the presence of *E*. *faecalis*, *E*. *coli*, *S*. *agalactiae*, and *G*. *vaginalis* in females was significantly associated to reduced levels of vaginal lactobacilli, (iv) the presence of the group *E*. *faecalis*, *U*. *urealyticum*, and *M*. *hominis* in infertile couples was significantly associated to IVF negative outcome.

All the infertile couples enrolled in the study were asymptomatic for genital tract infections, but nearly half (46.3%) resulted positive to microbiological testing. As to our knowledge no data are available in the literature on the prevalence of asymptomatic genital tract infections in naturally fertile couples, it is difficult to estimate the impact of asymptomatic infections on fertility. The microbial species identified in our study are mostly colonizers of the male anterior urethra and coronal sulcus or the vaginal milieu [31,32], but are also responsible of urinary tract (UTI), genital and systemic infections. The prevalence of pathogens was different between males and females, with certain microbial species showing a clear predominance in males (*E*. *faecalis*, *S*. *haemolyticus*, Group F *Streptococcus*) or females (*M*. *hominis*, *Candida* spp., *G*. *vaginalis*, *T*. *vaginalis*). *E*. *faecalis* and *M*. *hominis*/*S*. *agalactiae* were the most frequently detected microbes in males and females, respectively. *M*. *hominis* is a common endosymbiont of *T*. *vaginalis*, which acts not only as a protective niche but also as a ‘Trojan horse’ to transmit the bacteria to the human genital tract [33]. In our case, although the prevalence of the protozoan in infected females was low (4%), 66.7% of the *T*. *vaginalis*-positive vaginal swabs also contained *M*. *hominis*. Out of all pathogens detected in infected couples, *E*. *faecalis* was the most common (32.6%) and was shown to adversely affect couple fertility.

Genital infection and inflammation can impact on male fertility in several ways, including deterioration of spermatogenesis, impairment of sperm functions, generation of ROS leading to DNA fragmentation or oxidative protein modification, production of sperm antibodies, and obstruction of the seminal tract [4]. The relationship between bacteriospermia, leukocytospermia and semen parameters in infertile men is still debated. Some studies showed a detrimental influence of microbial pathogens or/and leukocytes on semen quality [5,7,10,34–37], while others did not observe any effect [6,38,39]. In this study, 29.1% of semen specimens was positive to microbiological analysis, which is in accordance with literature data reporting that rates of bacteriospermia in infertile men can fluctuate from 15 to 60% [5–7,36,39]. The most prevalent species was *E*. *faecalis*, which was significantly associated to reduced motility and altered morphology of spermatozoa, suggesting that enterococci may have a direct negative influence on semen quality as previously published [5,6,36]. In contrast, significant sperm abnormalities were not observed in *E*. *coli*- and *S*. *agalactiae*-positive samples, the second and third most commonly isolated species. Some authors have shown that *E*. *coli* induces alterations in human spermatozoa, resulting in reduced motility, altered acrosomal function, and decreased vitality [5,35,40]. Nonetheless, the fact that those data mostly originate from *in vitro* studies based on large bacterial concentrations that will unlikely be reached during *in vivo* infection, may explain our negative result on *E*. *coli*. Leukocytospermia is generally considered as a marker of inflammation with a poor diagnostic value for genital tract infections [6,7,41]. Several factors independent of infectious challenges have been associated to elevated seminal leukocytes, including ageing, medications, smoking, alcohol and drug abuse [7,8]. In our study, leukocytospermia was not significantly associated with the presence of microbial pathogens in semen (*p* = 0.09), as evidenced by the fact that 79% of leukocytospermic samples were microbiologically negative as reported before [42]. This finding, which is not unexpected [6,7,41,42,43], probably indicates that the presence of potential pathogens in semen does not necessarily lead to a full-fledged inflammatory response with substantial leukocyte recruitment. The hypothesis may be especially valid in asymptomatic patients as those enrolled in the present study. Moreover, the time of semen collection could be another relevant factor to explain our data since bacteria and leukocytes may not be present simultaneously in semen samples as described [44].

BV, AV and abnormal vaginal flora (decrease/absence of lactobacilli) have been reported to affect pregnancy rate and outcome [24]. A recent systematic review and meta-analysis showed that BV is associated with female infertility, preclinical pregnancy loss and preterm birth, although it does not seem to impact on conception rates [21]. Prevalence of BV in infertile women varies considerably from 10% to 45% depending on the study population and diagnostic criteria [21,25,45,46]. Diagnosis of BV can be performed based on clinical, microscopic, and (cultural and/or molecular) microbiological criteria [19]. The Nugent scoring system was the first standardized method founded on classification and enumeration of ‘morphotypes’ in Gram-stained vaginal smears [47]. Despite being long recognized as the gold standard for BV diagnosis, the Nugent system has several limitations [19]. In the current study, we chose a combined culture- and molecular-based diagnostic approach for detection and quantification of both pathogens and lactobacilli in genital swabs. Microbiological analysis showed that the presence of vaginal lactobacilli was generally associated to absence of genital pathogens; conversely, detection of *E*. *faecalis*, *E*. *coli*, *S*. *agalactiae*, and *G*. *vaginalis* was significantly associated with reduced levels of vaginal lactobacilli. *G*. *vaginalis* is strongly linked to BV [19], whereas *E*. *coli*, *S*. *agalactiae*, and *E*. *faecalis* are not typical BV-defining microorganisms but are instead common agents of UTI and AV. Absence of lactobacilli, besides being a key feature of BV, has also been correlated to AV [22,48] and recurrent UTI by *E*. *coli* [49] and other uropathogens [50], underlining the protective function exerted by lactobacilli against urogenital infections. In contrast, the presence of *M*. *hominis*, another recurrent species in BV, was not significantly associated to decreased numbers of vaginal lactobacilli in this study population. It should also be noted that a small percentage (18.6%) of females negative to microbiological testing had low amounts of lactobacilli, indicative of either a healthy microbiota not dominated by *Lactobacillus* spp. [18] or a transitional stage in the dynamic shifts of the vaginal microbial ecosystem [51]. The outcome of IVF has been associated with the composition of the vaginal microbiota on the day of ET [52], and a microbiota exclusively composed by lactobacilli is considered the most promising scenario for successful IVF [53]. In our case, only 12.8% of patients with reduced quantity of lactobacilli belonged to the IVF+ couples, yet again emphasizing the key role played by a *Lactobacillus*-dominated microbiota for a positive outcome of IVF.

In the current study, IVF negative outcome was slightly more elevated in infected than non-infected couples, but differences were not significant. However, specific pathogens were more frequently found in IVF- couples, and hence a definite infectious group (*E*. *faecalis* and/or *U*. *urealyticum* and/or *M*. *hominis*) was identified that significantly correlated with IVF failure (*p* = 0.02). It should be noted that *G*. *vaginalis* was initially included in the group (*p* = 0.031), in accordance with a recent prospective study reporting the association of *G*. *vaginalis* and *A*. *vaginae* with low pregnancy rates in IVF patients [25]. Identification of a group of infectious agents, rather than a single microbial species, is not unexpected, as the above pathogens can be found as agents of polymicrobial genital tract infections [19,50,54]. *M*. *hominis* has been shown to synergistically cooperate with *G*. *vaginalis* in BV [19,55] and is a frequent symbiont of *T*. *vaginalis* in trichomoniasis and BV [33,56], while *E*. *faecalis* can be detected in polymicrobial urogenital and biofilm-based infections [50]. Whether these microbes act as either independent or bystander pathogens in genital tract infections is not clear yet, however, increasing evidence is accumulating on their role in infertility, adverse pregnancy outcome, and post-partum complications [11,13,19,21,22,24,25,54,57]. Lower rates of fertilization, implantation and clinical pregnancy were observed in couples undergoing ICSI that were positive to *E*. *coli*, *E*. *faecalis*, *S*. *agalactiae*, and *Staphylococcus* spp. [52,58]. Detection of genital *Mollicutes* in couples undergoing IVF was also associated to reduced pregnancy rates [59,60] and increased miscarriages [61]. To this regard, it is interesting to note that all the couples of this study infected by *M*. *hominis* had a negative IVF outcome. The present work shed light on three specific genital tract pathogens as predictive infectious markers for poor outcome of IVF, emphasizing the importance of microbiological testing of infertile couples for *E*. *faecalis*, *U*. *urealyticum*, and *M*. *hominis* prior to IVF procedures.

In conclusion, infertility is a multi-factorial and multi-faceted clinical condition which poses a profound economic and psychological burden on affected couples and high costs on the healthcare system. In this study, we have identified an infectious group that significantly correlated as an independent risk factor to infertility and negative outcome of IVF. However, the causes of IVF failure often remain unknown. A joint effort between clinical microbiologists, infectious diseases and reproductive medicine specialists is desirable to produce consensus guidelines on testing for pathogens associated to infertility and assessment of both microbiological and clinical outcomes before ART treatment. Improved management of genital tract infections in infertile couples may be helpful to increase pregnancy rates, reduce the total number of treatment cycles and possibly enhance first level fertility approaches with beneficial effects on couple well-being and healthcare costs.

## Supporting information

S1 Table Primers used for Real-Time PCR. List of primers and probes used to identify non-cultivable pathogens in genital tract samples.
(DOCX)
