# Supplementary material for: Impact of asymptomatic genital tract infections on in vitro Fertilization (IVF) outcome
Source: PLoS One. 2018 Nov 16;13(11):e0207684. doi: 10.1371/journal.pone.0207684 (PMC6239332; doi:10.1371/journal.pone.0207684)
Supplement: S1 Table — List of primers and probes used to identify non-cultivable pathogens in genital tract samples. (DOCX) [file pone.0207684.s001.docx]

| **Microorganism** | **Primer/probe ^a^** | **Sequence (5’ -3’)** | **Target gene** | **Reference ^b^** |
| --- | --- | --- | --- | --- |
| *C. trachomatis* | CTplas-F | CAGCTTGTAGTCCTGCTTGAGAGA | *omcB* | Pickett *et al*., 2005 |
|  | CTplas-R | CAAGAGTACATCGGTCAACGAAGA |  |  |
|  | CTplas-Tp | *6FAM-*CCCACCATTTTTCCGGAGCGA-*TAMRA* |  |  |
| *N. gonorrhoeae* | porA-F | CCGGAACTGGTTTCATCTGATTAC | *porA* | Hjelmevoll *et al*., 2006 |
|  | porA-R | GGTTTCAGCGGCAGCATTCAAT |  |  |
|  | porA-Tp | *6FAM*-AGTAGCAGGCGTATAGGCGGACTT-*TAMRA* |  |  |
| *M. hominis* | VM-F | TTCATGTACTACTAACTGTTTAGCTC | *gap* | This work |
|  | VM-R1 | TGGAGCATCTTGTAATCTTTGGT |  |  |
|  | VM-Tp | *6FAM-*CCTATTGCCAACGTATTGG*-MGB* |  |  |
| *U. urealyticum* | UUureF | GATCACATTTCCACTTATTTGAAACA | *ureB* | Mallard *et al*., 2005 |
|  | UUureR | AAACGACGTCCATAAGCAACTTTA |  |  |
|  | UUure2MGB | *6FAM-*AAACGAAGACAAAGAAC*-MGB* |  |  |
| *T. vaginalis* | L23861-F | AAGATGGGTGTTTTAAGCTAGATAAGGT | *btub* | Schirm *et al*., 2007 |
|  | L23861-R | CGTCTTCAAGTATGCCCCAGTAC |  |  |
|  | L23861-Tp | *6FAM* -CCGAAGTTCATGTCCTCTCCAAGCGT-*TAMRA* |  |  |
| HSV | HSV2K-F | TACCACATTCAGCCGAGCC | US6 | This work |
|  | HSV2K-R | CCATGCGATACCAGGCGAT |  |  |
|  | HSV2K-Tp | *6FAM*-CAGGTTGTACGTGT-*MGB* |  |  |

**Table. Primers used for Real-Time PCR.**

^a^ Tp, Taqman probes. *6FAM*, 6-carboxylfluorescein; *TAMRA*, tetramethylrhodamine; *MGB*, minor grove binder.

^b^ - Pickett MA, Everson JS, Pead PJ, Clarke IN. The plasmids of *Chlamydia trachomatis* and *Chlamydophila pneumoniae* (N16): accurate determination of copy number and the paradoxical effect of plasmid-curing agents. Microbiology. 2005;151:893-903.

- Hjelmevoll SO, Olsen ME, Sollid JU, Haaheim H, Unemo M, Skogen V. A fast real-time polymerase chain reaction method for sensitive and specific detection of the *Neisseria gonorrhoeae* *porA* pseudogene. J Mol Diagn. 2006;8:574-81.

- Mallard K, Schopfer K, Bodmer T. Development of real-time PCR for the differential detection and quantification of *Ureaplasma urealyticum* and *Ureaplasma parvum*. J Microbiol Methods. 2005;60:13-9.

- Schirm J, Bos PA, Roozeboom-Roelfsema IK, Luijt DS, Moller LV. *Trichomonas vaginalis* detection using real-time TaqMan PCR. J Microbiol Methods. 2007;68:243-7.
